# Hypnotism and Crime

**Published:** 1898-09-17

**Authors:** 


### The Hospital

A Journal of

The Medical Sciences and Hospital Administration.

Vol. XXIV.—No. 625.] Edited by Sir Henry Burdett, K.C.B., and Solomon C. Smith, M.D., M.R.C.P. [Sept. 17, 1898.

### Hypnotism and Crime.

Those who practice hypnotism find themselves in a difficult position. Of the reality of the condition which goes by the name "hypnotism" there can be no doubt; of its power there can be no question. Under its influence the sense of pain can be abolished, and the subject can be induced to do strange actions, which he entirely forgets in the waking state—actions so strange that he certainly would not have done them had his will retained its normal command. The power of hypnotism has to be admitted—has even to be claimed—if it is to maintain its position. Yet, on the other hand, if the hypnotists would gain the confidence of the public and of the medical profession, they are almost driven to maintain that the hypnotic state cannot be made use of for criminal purposes. The task is a difficult one; nay, the contradiction is so obvious that we must be excused if we express our belief that it is impossible to prove that hypnotism is powerless for evil without, at the same time, undermining many of the claims which have been made in regard to its capacity for doing good.

According to a certain school all hypnotism is suggestion. The attention is concentrated, a suggestion is made, it is acted upon automatically, and that is all. Nothing can be more simple. But those who adopt this view are bound to explain what is to prevent an immoral suggestion becoming as effective as an innocent or merely absurd one. It is said, of course, that no one will do an act which is contrary to his moral nature, and it is easy to believe that the habitual tendencies of the individual will still have considerable influence, even after the hypnotic state has been induced. That, however, hardly touches the question, for many people remain virtuous in deference to law rather than to moral nature, and we would like to know what safeguard there would be if crime were suggested to the habitual criminal.

Dr. Milne Bramwell, speaking at Edinburgh, held that volition is retained during hypnotism. He had questioned profound somnambules during hypnosis as to their own mental condition, and all practically gave similar replies, namely, that when in the hypnotic state they knew that they were hypnotised, and retained completely the sense of their personal identity and relationship with the outside world. He also said that, even in profoundly lethargic conditions, in which the subjects might be supposed to be insensible to external conditions, they would resist suggestions which were displeasing to them.

This may be so in regard to his cases, and we do not wish to speak too positively on the subject; but this much has to be remembered that if we admit that suggestion, as ordinarily practised, can influence not only action but belief; we must not be too ready to accept the statements of hypnotic subjects made while under hypnotic influence. Rather do we feel driven to go back to common knowledge of what has been done again and again in the most public manner, and to deduce from it what might occur. This point has been urged by Dr. Mercier with considerable force. He shows that the connection between hypnotism and crime would by no means be negatived even if it could be proved that it was impossible to induce a patient to commit a crime as a crime. "The point is and the fear is," he says, "that a patient may be made to commit a crime which has been suggested to him as a purely innocent act. The common exhibition of the itinerant mesmerist is to make a patient eat a tallow candle on the suggestion that he is eating a stick of celery, or to drink soap and water under the suggestion that he is drinking beer. Why then might not a butcher cut the throat of a child under the suggestion that he is cutting the throat of a sheep? Why should he not be made to poleaxe a man under the suggestion that he is poleaxing a bullock? Or, to put a more probable and more practical case, why should not a man be induced to sign an important document under the suggestion that he was signing something of a totally different character and of no importance?" We do not think that these questions have been answered. It certainly is no answer to show that certain individuals have retained throughout the hypnotic trance a certain degree of volition, and have been able to exercise a certain amount of discretion. What about eating the tallow candle? At the same time we are quite ready to admit that there is a good deal in hypnotism that is not easily explained by suggestion, and that it is possible that some of the therapeutic efficacy of the process may be due more to the psychological state induced than to the mere suggestions made by the hypnotiser. Dr. Bramwell, indeed, goes so far as to say that "suggestion no more explains the phenomena of hypnotism than the crack of a pistol explains a boat race. Both are simply signals—mere points of departure, and nothing more." If that is so, might we not invent some other signal, and could we not find some other "point of departure" from which to obtain the asserted benefits of hypnotism.

